# Mining the Unmapped Reads in Bovine RNA-Seq Data Reveals the Prevalence of Bovine Herpes Virus-6 in European Dairy Cows and the Associated Changes in Their Phenotype and Leucocyte Transcriptome

**DOI:** 10.3390/v12121451

**Published:** 2020-12-16

**Authors:** Laura Buggiotti, Zhangrui Cheng, D. Claire Wathes, GplusE Consortium

**Affiliations:** Department of Pathobiology and Population Sciences, Royal Veterinary College, Hawkshead Lane, Hatfield AL9 7TA, UK; lbuggiotti@rvc.ac.uk (L.B.); zcheng@rvc.ac.uk (Z.C.)

**Keywords:** bovine herpes virus-6, Gammaherpesvirinae, BoHV-4, dairy cows, RNA-Seq, mastitis, endometritis, leukocyte, interferon, ISGylation

## Abstract

Microbial RNA is detectable in host samples by aligning unmapped reads from RNA sequencing against taxon reference sequences, generating a score proportional to the microbial load. An RNA-Seq data analysis showed that 83.5% of leukocyte samples from six dairy herds in different EU countries contained bovine herpes virus-6 (BoHV-6). Phenotypic data on milk production, metabolic function, and disease collected during their first 50 days in milk (DIM) were compared between cows with low (1–200 and *n* = 114) or high (201–1175 and *n* = 24) BoHV-6 scores. There were no differences in milk production parameters, but high score cows had numerically fewer incidences of clinical mastitis (4.2% vs. 12.2%) and uterine disease (54.5% vs. 62.7%). Their metabolic status was worse, based on measurements of IGF-1 and various metabolites in blood and milk. A comparison of the global leukocyte transcriptome between high and low BoHV-6 score cows at around 14 DIM yielded 485 differentially expressed genes (DEGs). The top pathway from Gene Ontology (GO) enrichment analysis was the immune system process. Down-regulated genes in the high BoHV-6 cows included those encoding proteins involved in viral detection (*DDX6* and *DDX58*), interferon response, and E3 ubiquitin ligase activity. This suggested that BoHV-6 may largely evade viral detection and that it does not cause clinical disease in dairy cows.

## 1. Introduction

Cattle populations are exposed to a wide variety of acute and chronic diseases caused by pathogens derived from many different members of the animal kingdom including viruses, bacteria, protozoa, Platyhelminthes, and nematodes. Some of these are zoonotic (e.g., Cryptosporidiosis) [1]. Others (e.g., bovine viral diarrhea) reduce immunity, thus making cattle more susceptible to other diseases and increasing antibiotic usage [2]. Many bovine diseases are endemic in the population where they cause financial losses through mortality, decreased outputs of milk and meat, reductions in fertility, premature culling, and costs of veterinary treatment and prevention strategies [3]. The complexity, variety, and interaction of cattle pathogens have therefore made it difficult to fully understand their impact on the animals’ health and which effective measures to undertake to achieve eradication, or at least acceptable control, at both the herd and national levels. At present, bovine pathogen diagnostic procedures are based on a variety of methodologies including post mortems, antibiotic susceptibility testing, serological antibody profiles, targeted PCR, histopathology, and microbiological culture. These techniques may be time-consuming and expensive, and they may vary in their sensitivity and specificity. Furthermore, in many cases, each individual disease requires a separate test, and latent infection among susceptible animals may render serology alone inconclusive evidence of current disease.

For many diseases, however, distinctive transcripts of the pathogen RNA may be present in blood samples of the host [4,5]. Recent developments in RNA sequencing technology mean that it is now possible to perform highly sensitive whole transcriptome sequencing with the accurate detection of only a few transcript copies per cell [6]. The RNA reads produced from next-generation sequencing are usually mapped against a reference genome using alignment algorithms [7]. Following this process, a significant number of reads remain unmapped due, in part, to sequencing errors and sequence variants. These unmapped reads were previously considered as “junk.” Recent work has, however, shown that they also contain transcripts derived from, and specific to, a variety of pathogens [8,9,10]. Using appropriate bioinformatic pipelines, it is therefore possible not only to look for evidence of particular microbes but also to discover novel viruses in circulation.

We used this approach to mine for viral transcripts in blood samples collected from six European dairy herds. This analysis revealed the widespread presence of bovine herpes virus 6 (BoHV-6). This belongs to the subfamily Gammaherpesvirinae within the genus *Macavirus*, which includes viruses that infect lymphoid cells of both domestic and wild ruminants [11,12]. BoHV-6 was first isolated from the leukocytes of cattle with lymphosarcoma [13] and was originally known as bovine lymphotropic herpesvirus [11]. It has since been reported in dairy cow populations in a variety of countries including the USA [11,12], Canada [14], New Zealand [15], United Kingdom [16], Belgium [17], and Poland [18]. Care in diagnosis is necessary, however, as BoHV-6 shares 65% sequence homology with BoHV-4 [18], and infected cows were found to test positive in a commercial ELISA directed against BoHV-4 [15].

There is much that remains unknown about this virus, including the mode of transmission and its relevance, if any, to clinical disease. The cows used in our study were part of GplusE, an FP7-Project funded by the European Union (http://www.gpluse.eu) to investigate links between the phenotypes and genotypes of commercial dairy cows in order to improve their breeding and management. Extensive phenotype and blood transcriptome data from cows in early lactation were therefore already available. This enabled us to investigate the associations between the presence of BoHV-6, clinical disease, metabolic status, and gene expression in circulating leucocytes in relation to the viral load detected, as described in the present paper.

## 2. Materials and Methods

### 2.1. Animals

The animals contributing to this study were enrolled as part of the GplusE study. All procedures had local ethical approval and complied with the relevant national and EU legislation under the European Union (Protection of Animals used for Scientific Purposes) Regulations 2012 (S.I. No. 543 of 2012). They included Holstein Friesian cows from six research herds each in different EU countries (Aarhus University, Denmark; University College Dublin Lyons Research Farm, University College Dublin, Ireland; Agri-Food and Biosciences Institute, Northern Ireland, UK; Leibniz Institute for Farm Animal Biology, Germany; Walloon Agricultural Centre, Belgium; and Consiglio per la Ricerca in Agricoltura e l’Analsi dell’Economia Agraria, Italy). Details of the management of each herd and phenotypic data collection were described previously [19]. All sampling and diagnostic methods were performed according to standard operation procedures agreed within the consortium [19,20]. All chemicals were obtained from Sigma Aldrich (Gilligham, Kent, UK) unless otherwise stated.

### 2.2. Leukocyte Collection and RNA Preparation 

Blood samples were collected from each cow included in this study (*n* = 195) at around 14 days after calving (days in milk—DIM) into Tempus blood collection tubes (Thermo Fischer, Loughborough, UK). These were shaken vigorously for 15–20 s immediately upon collection, and then they were frozen and stored at −80 °C. Some repeat samples (*n* = 17) taken at 35 DIM were also used. All samples were shipped frozen to the Royal Veterinary College, Hatfield, UK, for extraction. Tempus tubes were thawed at room temperature before decanting the contents into 50 mL conical tubes. Whole blood extraction (containing leukocytes) used Tempus Spin RNA isolation Kits (Thermo Fischer, Loughborough, UK) following the manufacturer’s instructions. RNA quantity and integrity were assessed using an Agilent BioAnalyzer 2000 (Agilent Technologies UK Ltd., Cheadle, UK) and Agilent RNA 6000 Nano Kit (Agilent). RNA measurements were also validated using a NanoDrop 1000 (Thermo Fischer).

### 2.3. RNA-Sequencing

RNA-Seq libraries were prepared from the whole blood samples using an epMotion liquid handling workstation (Eppendorf, Hamburg, Germany) with the Illumina TruSeq Stranded Total RNA Library Prep Ribo-Zero Gold kit (Illumina, San Diego, CA, USA) at the University of Liege (GIGA Research Facility, Liege, Belgium). Pooled cDNA libraries were sequenced on an Illumina NextSeq 500 sequencer (4 lanes multiplexed) at 75 nucleotide length single end reads to reach an average of 30 million reads per sample.

### 2.4. Transcriptomics Pipeline

Reads were trimmed or removed according to base quality using Trimmomatic v. 0.36 [21] and the following setting (*SLIDINGWINDOW:4:25 MINLEN:30 LEADING:3 TRAILING:3*). The quality of raw and trimmed FASTQ files was assessed with FastQC (http://www.bioinformatics.babraham.ac.uk/projects/fastqc/). *Bos taurus* assembly (ARS_UCD1.2) and its corresponding gene set were used as reference to map reads using the splice aware aligner HISAT2 [22]. SAM files were converted to BAM files and coordinate sorted with SAMtools [23]. Picard Tools (http://picard.sourceforge.net) were used on the BAM files to remove PCR duplicates, add read group information, sort by chromosome, and create indexes. Reads per protein coding gene were counted with StringTie [24], and expression values were normalized as reads per kilobase of transcript per million mapped reads (RPKM).

### 2.5. Pipeline for Analysing Unmapped Reads

The conversion of the RNA-Seq data from FASTQ format to the unaligned BAM format was accomplished using the ‘FastqToSam’ command (http://broadinstitute.github.io/picard/). Afterwards, the unaligned BAM was passed into the ‘PathSeqPipelineSpark’ pipeline, a sequence-based computational subtraction approach distributed in the GATK genome analysis toolkit v4.1.6 [25]. The viral references for mapping were created using representative genomes (12,148 total genomics entries) from the National Center for Biotechnology Information (NCBI, Bethesda, MD, USA), as well as the PathSeq reference creation tools. The host reference for the mapping was *Bos taurus* (ARS-UCD1.2). Using these mapping results, the inferred viral abundance profiles in each sample were assembled by PathSeq using default parameters. Included in these profiling data were the raw read counts, adjusted scores, and normalized scores (compositional data from the adjusted scores that represent inferred relative abundance) for taxa within each taxonomic classification (genera and phyla). The PathSeq score, based on the number of reads that align with taxon references, indicates the amount of evidence that a taxon is present in a given sample. Samples with a PathSeq score for BoHV-6 ≥ 1 were initially taken as positive. As the main focus of this study was to compare the phenotypes of cows with a low or high BoHV-6 load, we subsequently categorized the samples as low BoHV-6 (PathSeq score of 1–200) or high BoHV-6 (PathSeq score > 200) for subsequent analysis.

### 2.6. Weights and Body Condition Scores

Sample and production data were obtained between calving and 50 DIM. Body weights (BWs) were recorded twice weekly using weigh scales. Daily individual dry matter intake (DMI) was recorded using electronic feeding systems. The body condition score (BCS) was recorded according to a common protocol at 14 ± 1.8 and 35 ± 1.8 DIM (mean ± SD) using a five-point scale, with each score subdivided into quarters so that, in effect, it functioned as a 20-point scale [26].

### 2.7. Analysis of Blood Metabolites and IGF-1 

Blood samples were taken at around 14 DIM into both serum and heparin tubes by jugular or coccygeal venipuncture, separated by centrifugation, and stored at −20 °C for subsequent analysis. The laboratory analysis of blood metabolites was performed at the Department of Animal Science, Aarhus University, Denmark. Urea and cholesterol were determined in plasma according to standard procedures using an ADVIA 1800^®^ Chemistry System auto-analyzer (Siemens Medical Solutions, Tarrytown, NY, USA). Glucose, non-esterified fatty acids (NEFA), β-hydroxybutyrate (BHB), and fructosamine were determined as described previously [27]. Intra- and inter-assay co-efficients of variation CV were, in all cases, below three and four percent, respectively, for low and high control samples. Concentrations of IGF-1 were determined in serum by a radioimmunoassay at University College Dublin, Ireland, following acid–ethanol extraction using the previously described method [28]. Intra-assay CV were 12.4%, 7.5%, and 9.9% for low, medium, and high control samples, respectively. The corresponding inter-assay CV were 7.8, 3.9, and 9.4%. The sensitivity of the assay, defined as the lowest concentration detectable, was 4 ng/mL.

### 2.8. Milk Sample Collection, Production Data, and Analysis for Metabolites and Enzymes

All cows were milked twice daily, and their daily yields were recorded. Milk samples containing 0.02% bronopol as a preservative were collected from consecutive morning and evening milkings twice weekly between seven and 49 DIM, stored at 4 °C, and subsequently analyzed by FT-MIR for the composition of protein, fat, lactose, and somatic cell count (SCC) for milk quality testing. Additional morning milk samples (two × 8 mL) were collected twice weekly and stored at −18 °C prior to shipping to the Department of Animal Science, Aarhus University, Denmark, for analysis. Fluorometric end point analyses were used to determine milk glucose and glucose-6-phosphate, uric acid [29], isocitrate [30], and BHB [31]. Urea was determined by spectrophotometry [32]. The enzymes lactate dehydrogenase (LDH) (EC. 1.1.1.27) and *N*-acetyl-β-D-glucosaminidase (NAGase) (EC 3.2.1.30) were analyzed by fluorometric assays [33]. Intra- and inter-assay CV were, in all cases, below five and eight percent, respectively, for low and high control samples.

### 2.9. Health Records

Health information on study cows was obtained from herd records. Clinical mastitis was diagnosed using standard methods based on daily observations for abnormal changes in milk appearance (e.g., flakes and clots), quality, milk yield, and mammary inflammatory responses (redness, swelling, heat, or pain). The cows were subsequently categorized into three groups. Healthy cows were defined as having an SCC <100,000 cells/mL milk with no clinical symptoms. Sub-clinically mastitic cows were defined as having an SCC between 100,000 and 400,000 cells/mL milk with no apparent clinical symptoms. In the cows diagnosed as having clinical mastitis, their SCC was >400,000 cells/mL milk, and they showed some of the above clinical symptoms.

Cows with uterine disease were defined as having metritis or endometritis as follows: (1) puerperal metritis, an abnormally enlarged uterus and a fetid watery red-brown uterine discharge that is associated with signs of systemic illness and fever >39.5 °C within 21 days after calving; (2) clinical metritis, as for (1) but without systemic illness; and (3) clinical endometritis, the presence of purulent (>50% pus) uterine discharge detectable in the vagina >21 days after calving or mucopurulent discharge, containing approximately 50% pus and 50% mucus, detectable in the vagina >26 days after calving [34].

A uterine cytobrush sample (Minitube, Minitüb GmbH, Tiefenbach, Germany) was also taken from every cow in the study at around 14 and 35 DIM to evaluate endometrial cytology, as previously described [35]. A double guarded cytobrush was guided manually through the cervix into the uterus, the inner guard was extruded from the outer guard, and the brush was rotated gently against the endometrial wall. The brush was then withdrawn into the inner guard and removed. Slides for cytological examination were prepared by rolling the cytobrush onto a clean glass microscope slide and fixing the sample with Fisherbrand™ CytoPrep™ Cytology Fixative (Fishers Scientific, Blanchardstown, Ireland). Fixed slides were sent to the UCD School of Veterinary Medicine, University College Dublin, Ireland. for processing and stained with modified Giemsa stain. Cytological assessment was performed by counting at 400× magnification (Leitz Labourlux-S, Wetzlar, Germany) to determine the ratio between polymorphonuclear neutrophils (PMNs), uterine epithelial cells (UECs), and the percentage of neutrophils (%PMN), averaging 10 counts per slide. The mucus content of the vagina was also evaluated for both appearance and smell, as described previously [36]. Briefly, the cow’s vulva was thoroughly cleaned with a dry paper towel; a clean, lubricated, gloved hand was inserted through the vulva; the walls of the vagina and the external cervical os were palpated; and the mucus contents of the vagina were manually withdrawn. The vaginal mucus was assessed for color and proportion and volume of pus, and then it was scored as: 0 for clear or translucent mucus, 1 for mucus containing flecks of white or off-white pus, 2 for <50 mL exudate containing over 50% white or off-white mucopurulent material, or 3 for >50 mL of exudate containing purulent material, usually white or yellow but occasionally sanguineous. The vaginal mucus was also assessed for odor and given a score of 0 for normal or 1 for fetid.

### 2.10. Statistical Analysis

RNA-Seq gene expression data were analyzed using Partek Genomics Suite (PGS) Software V7.1 (Partek Incorporated, Chesterfield, MO, USA) after the log2-transformation of the RPKM values. The effect of different herds on gene expression was removed using the function built in Partek Genomics Suite V7.1 (Supplementary Figure S1). This function calculates the variation attributable to herd then adjusts the original values to remove the variation. Differences between the cows based on low or high BoHV-6 scores were analyzed by Student’s *t*-test following the removal of the herd effect. BH adjustment was used, and statistical significance was considered at *p* < 0.05. Differentially expressed genes (DEGs) that met the selection criteria BH adjustment *p* < 0.05 and fold change >1.2 were taken forward for Gene Ontology (GO) enrichment analysis using Partek Genomics Suite V7.1. with the ARS_UCD1.2 genome and Fisher’s exact test with a BH adjustment at *p* < 0.05.

The data derived from blood and milk metabolites are summarized as mean ± SD. Differences between the cows with high and low BoHV-6 scores were determined with Mann–Whitney U test using SPSS V26 (SPSS Inc., Chicago, IL, USA). Statistical significance was considered where *p* < 0.05.

## 3. Results

### 3.1. BoHV-6 Pathseq Scores

**Figure 1:**
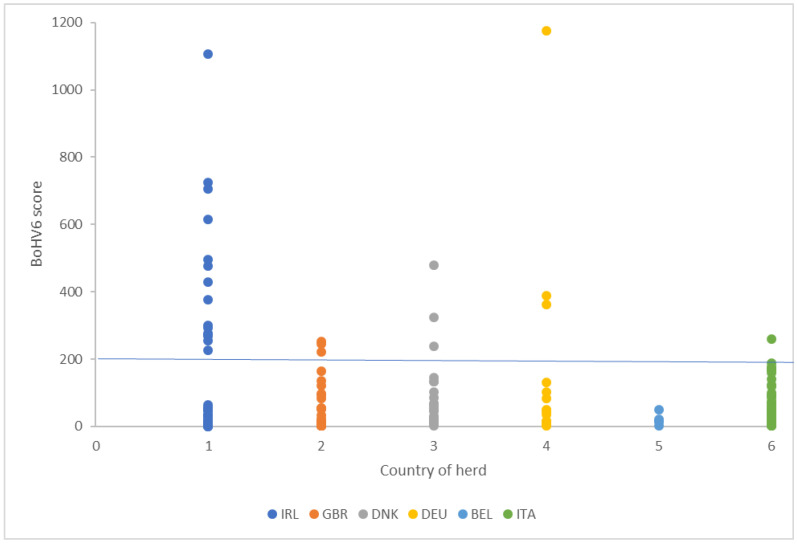
Individual bovine herpes virus-6 (BoHV-6) scores in dairy cows from 6 herds by country of origin (IRL, Ireland; GBR, United Kingdom; DNK, Denmark; DEU, Germany; BEL, Belgium; and ITA, Italy). The horizontal line set at 200 was taken to distinguish cows with a low (1–200) or high (>200) score.

RNA-Seq data were available from 195 cows. Of these, 163 samples (83.5%) mapped to the BoHV-6 genome with a score of between 1 and 1175, whereas in 32 cows (16.4%), the unmapped bovine reads did not map to the BoHV-6 genome but did detect other pathogens with low frequencies. Using data from the 163 cows with a score ≥1, the average scores for the six herds ranged from 18 ± 16 to 215 ± 269 (mean ± SD). Based on the score distribution (Figure 1), a score of 1–200 or >200 was used to denote cows with low or high BoHV-6 values. The proportion of animals scoring above the 200 threshold ranged from 0% to 44% between herds. Out of 163 cows in the study, 15% were classified as having a high BoHV-6 score (Table 1).

**Figure 2:**
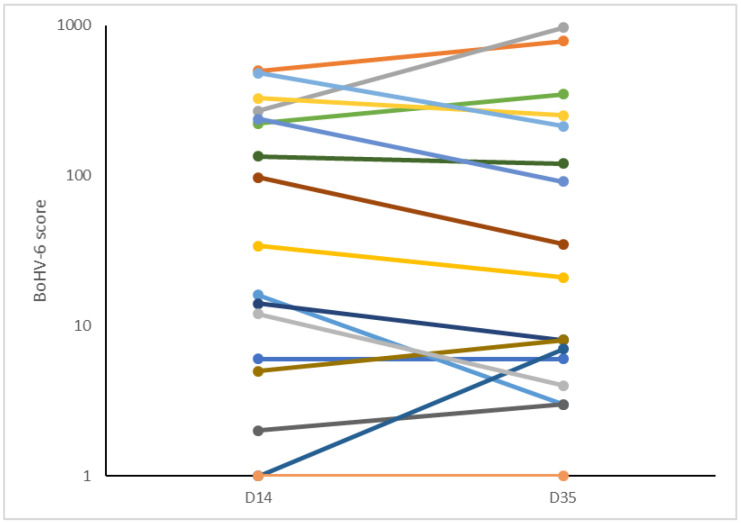
Log10 BoHV-6 scores in 17 cows in which repeat samples of leukocytes were analyzed at both 14 and 35 days in milk (DIM).

Seventeen cows had repeat samples taken at both 14 and 35 DIM. Overall, there was no change in the score between these two time points (BoHV-6 scores: 14 DIM: 138 ± 41; 35 DIM: 168 ± 69—mean ± SEM). The score stayed the same (within 10 units) in seven cows, it increased slightly in three, and it decreased in seven (Figure 2). Only one cow changed classification from high to low, with the score decreasing from 238 to 91.

BoHV-6 scores were also analyzed according to the lactation number of the cows as follows: lactation 1: score of 101 ± 120, with *n* = 21; lactations 2–3: score 83 ± 145 with *n* = 96; lactations 4–7: score of 142 ± 228 with *n* = 44 (mean ± SD). There was, therefore, no difference in the BoHV-6 score between lactations.

### 3.2. BoHV4 Pathseq Scores

The RNA from the same 163 BoHV-6-positive cows was also analyzed for the presence of BoHV-4 using the same PathSeq technique. Counts were only detected in eight cows, and these were all very low (score 2.1 ± 2.1, mean ± SD, and range 1–7).

### 3.3. Phenotype Comparisons of High and Low BoHV-6 Score Cows

Data from the Belgium and Italian herds were omitted at this point because they included zero and one cow, respectively, with a high BoHV-6 score, and their inclusion unbalanced the analysis. A comparison of the cow weights, BCS, and milk production over the start of lactation (first 50 DIM) between the remaining cows with high and low BoHV-6 scores is summarized in Table 2. The BCS was slightly higher in the high BoHV-6 score cows at both 14 and 35 DIM. No other differences in these parameters approached statistical significance, although it is interesting to note that the high BoHV-6 cows had a numerically higher weight but a lower dry matter intake.

The results of the metabolite analysis are summarized in Table 3. This shows that cows with a high BoHV-6 score had significantly lower circulating concentrations of IGF-1, urea, and fructosamine in early lactation, but there were no differences in plasma levels of BHB, NEFA, glucose, or cholesterol between the two groups. In milk, the concentrations of glucose-6-phosphate, uric acid and urea were all lower in the high BoHV-6 score cows, but the BHB concentration was higher. The enzyme LDH was significantly lower in the high BoHV-6 cows, with a similar trend (*p* = 0.07) for milk NAGase.

Records on mastitis were available from 82 low BoHV-6 cows and 24 high BoHV-6 cows. The low score cows had 10 cases of clinical mastitis diagnosed in the first 50 DIM compared with one high BoHV-6 cow (12.2% vs. 4.2% of cows, respectively). The SCC value in the milk sample taken closest to the blood sample used for the RNA-Seq analysis was also evaluated. For the low BoHV-6 cows, 63 were classified as healthy (SCC < 100,000 calls/mL; 76.8%), 12 were classified as having sub-clinical mastitis (SCC 100,000–400,000 cells/mL; 14.6%), and seven were classified as having clinical mastitis, SCC > 400,000 cells/mL; 8.5%); in comparison, the high BoHV-6 cows comprised 20 that were healthy (83.3%), two that had sub-clinical mastitis (8.3%), and two that had mastitis (8.3%). None of these differences in frequency were significant.

The records relating to the incidence of uterine disease were also compared between the two groups of cows and are summarized in Table 4.

The overall incidence of metritis and endometritis combined for the four herds with a significant proportion of BoHV-6-positive cows was 59/97 (60.8%). This was slightly lower than the incidence in the two herds that had little or no BoHV-6 present, which had 32/48 (66.7%) of cows diagnosed with uterine disease. In the four herds, there was no difference in the proportion of cows diagnosed with clinical uterine disease between the high and low BoHV-6 groups (χ^2^ = 0.47 and *p* = 0.49). Only one cow with a high BoHV-6 count also had BoHV-4 present, and there was no difference in the incidence of BoHV-4 between groups. This lack of difference between the groups was supported by measurements made to characterize the uterine discharge and to assess the proportion of PMN present in uterine cytobrush samples taken at both 14 and 35 DIM. In summary, these data failed to show any association between the presence of a high BoHV-6 score and the incidence of uterine disease.

### 3.4. Gene Expression in Leukocytes

**Figure 3:**
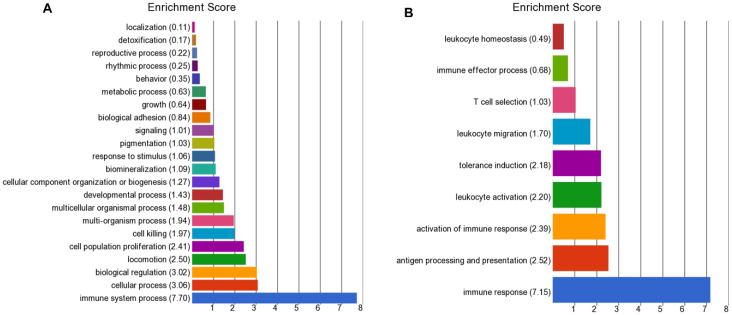
Gene Ontology (GO) enrichment analysis for (**A**) the differentially expressed genes (DEGs) by circulating leukocytes collected at around 14 days in milk between the low (1–200; *n* = 90) and high (>200; *n* = 24) BoHV-6 score cows and (**B**) sub analysis of the DEGs involved in immune system process.

For the gene expression analysis, a comparison was made between cows classified as having low (*n* = 90) or high (*n* = 24) BoHV-6 scores (1–200 or >200), using data from the four herds that included high score cows. A principal component analysis showed that the majority of samples from these two groups clustered separately (Supplementary Figure S2). Within the circulating leukocytes, there were 485 DEGs (*p* < 0.05 with BH adjustment and fold change FC > 1.25). Of these, 174 DEGs were up-regulated in the high BoHV-6 cows and 311 were down-regulated in comparison with the low BoHV-6 cows. The complete list of DEGs is provided in Supplementary Table S1. The GO enrichment analysis showed that the top five pathways with enrichment scores >2.4 were immune system process, cellular process, biological regulation, locomotion, and cell population proliferation (Figure 3A). Within immune system process, the top five sub-pathways with enrichment scores >2.18 were immune response, antigen processing and presentation, activation of immune response, leukocyte activation, and tolerance induction (Figure 3B).

The top 20 most significant DEGs are listed in Table 5. These were all more highly expressed in the leukocytes of high BoHV-6 cows. *AQP3* encodes a protein involved in the transport of water and non-ionic small solutes such as urea and glycerol across cell membranes. Three of the most significant genes are involved in protein folding within the endoplasmic reticulum (*TXNDC5*, *PDIA4*, and *XBP1*), while *CKAP4* encodes a protein that mediates the anchoring of the endoplasmic reticulum to microtubules. The top 20 most up-regulated genes in the high BoHV-6 cows based on fold changes are listed in Table 6. Three genes encoding fibrinogen chain members (*FGA*, *FGB*, and *FGG*) and the serum transport proteins albumin and transferrin were all up-regulated. The top 20 most down-regulated genes in the high BoHV-6 cows are also listed in Table 6. These included two genes located in close proximity on bta18 (*LOC513941* and *LOC527385*) that both encode proteins involved in the transport of cationic amino acids (arginine, lysine, and ornithine). Another gene, *SLC22A13*, that encodes a cation transporter involved in the transport of small molecules was also down-regulated. *NMUR2* encodes a receptor for neuromedin U, which plays an important role in the regulation of food intake and body weight. The insulin-like growth factor binding protein *IGFBP7* was up-regulated, but *IGFBP1* was down-regulated.

The differentially regulated genes involved in the immune system are listed in Table 7. More were down-regulated (*n* = 41) than up-regulated (*n* = 15) in the high BoHV-6 cows. The down-regulated genes included those encoding proteins identified as cytokines and chemokines, immunoglobulins, and related molecules and proteins contributing to the major histocompatibility complex. Of particular interest were key genes encoding proteins involved in the detection of viruses (*DDX6* and *DDX58*) and at least seven interferon-stimulated genes (ISGs) (*IFIT2*, *IFIT3*, *IFITM3*, *IFITM5*, *LOC112441484*, *LOC618409*, and *OAS1Z*). A further seven down-regulated genes were identified as having E3 ubiquitin ligase activity. Amongst the proteins encoded by the up-regulated genes, *XBP1* may increase the expression of viral proteins by acting as the DNA binding partner of a viral transactivator, and *C1QA* forms part of the first component of the serum complement system.

## 4. Discussion

Our results suggest that exploring whole blood transcriptomic unmapped reads from cows has huge and currently untapped potential to extract useful information regarding the presence of potential pathogens in domestic livestock. The RNA-Seq technology is highly sensitive and provides the precise measurement of levels of transcripts. It is now possible to detect many varied microbial sequences from a single sample, providing evidence that could be used for surveillance programs ranging from the individual herd to the national level. 

Within the six herds in our study, BoHV-6 was the most prevalent virus detected. The pathogen load is correlated with the PathSeq score, with a higher score providing more robust evidence that a taxon is indeed present based on the number of reads that aligned to the taxon reference. Walker et al. [25] developed the PathSeq tool within their genome analysis toolkit (GATK) and previously reported read counts of 1984 and 9882 for the human papillomavirus HPV-16 in mRNA and WGS libraries, respectively, derived from a patient with cervical cancer. The type of tissue used and its storage, the technique used to isolate the RNA, and the depth and quality of sequencing could all influence viral detection. In our study, all of these parameters were kept consistent. Our analysis was based on RNA-Seq data originally intended for traditional gene expression profiling, so pathogen detection was not the primary project aim and the methodology was not optimized with this in mind. Nevertheless, the highest PathSeq score for BoHV-6 was 1175, a similar figure to that reported in the HPV-16 study [25], and the reads gave repeatable values in pairs of samples taken from the same cow after a three week interval. For 16.4% of the cows in our study, the unaligned reads did not map to the BoHV-6 genome, although other pathogens were detectable in these samples at a low frequency. It is always difficult with this type of data to be completely confident at setting a value to discriminate animals that were definitely positive or negative. For an analysis of the phenotypic data, we therefore decided to omit the cows with no reads and to use a threshold of 200 to discriminate between basal levels and animals that had clearly elevated BoHV-6 scores (Figure 1). The gene transcription data provided sound evidence that leukocytes from animals in the high and low score groups showed real differences in viral detection and response pathways, thus validating this analytical approach.

Using a cut-off of 1, 85.5% cows tested positive for BoHV-6 across the six analyzed herds, with an incidence of high score >200 cows of between 0% and 44% between herds. This was in accordance with previous assessments. Within the USA, BoHV-6-specific DNA sequences have been found in peripheral blood mononuclear cells from 91% of adult samples tested [11] and 52–87% of cows in four dairies [37], while in Europe, 64% of 92 Polish dairy cows tested positive [18]. Rovnak et al. [11] also reported that 38% (*n* = 13) of two-week-old calves tested were positive for the presence of BoHV-6, suggesting that many animals become infected before or shortly after birth. Our study therefore supports previous data that this virus is indeed endemic in dairy cow populations worldwide.

The subfamily Gammaherpesvirinae preferentially infect lymphoid cells, where they establish latent infections and, for select viruses, cause malignant cell transformation. Reactivation can occur in immunosuppressed individuals. Within this subfamily, the genus *Macavirus* currently includes around nine species that infect domestic and wild ruminants and swine. BoHV-6 is most similar to alcelaphine herpesvirus 1 (AlHV-1) and ovine herpesvirus 2 (OvHV-2), sharing an average of 50% amino acid identity [12]. These two viruses cause asymptomatic infections in their reservoir hosts, which are wildebeest and sheep, respectively [38]. They can, however, also infect cattle and other ruminants, thus causing malignant catarrhal fever. This is a severe lymphoproliferative disease with a high mortality rate in which the activation and proliferation of latently infected T lymphocytes causes the inflammation and necrosis of a variety of internal organs [39,40]. With respect to BoHV-6, the virus has been detected in lymphoma cells, leucocytes, and peripheral blood mononuclear cells (PBMCs) [11,18]. As far as the authors are aware, no one has yet determined if it has a preference for B- or T-lymphocytes or monocytes, although the in vitro experimental infection of a bovine B-lymphocyte cell line has been demonstrated [11].

Though BoHV-6 was first isolated from the leukocytes of cattle with lymphosarcoma [13], most of the studies referenced above considered that the virus was not associated with disease. On the other hand, BoHV-6 has been isolated from the vaginal discharge of cows with postpartum metritis [15,16,17,41], an aborted fetus [14], and from buffaloes with lymphoproliferative disease [42]. Its association with uterine disease does, however, need to be interpreted with caution because BoHV-6 tested positive in a commercial ELISA directed against BoHV-4 [15]. BoHV-4 is cytopathic to endometrial epithelial and stromal cells [43], and there is accumulating evidence that it can act as a co-factor with established uterine bacterial pathogens such as *Escherichia coli* and *Arcanobacterium pyogenes* to promote the development of endometritis [44]. As cows infected with BoHV-4 appear more likely to have uterine disease after calving [45,46], there is a need to distinguish carefully which viruses are indeed present.

Our study provided the opportunity to examine associations between cows having a high BoHV-6 score with both milk yield and disease, as assessed over the first 50 days after calving. Over this time scale, there was no evidence that the virus had an adverse effect on milk production or quality. Indeed, LDH and NAGase, enzymes that act as inflammatory indicators of mastitis [33,47], were both lower in the milk of high BoVH-6 cows. This was in accordance with the slightly lower proportion of high BoHV-6 cows with a medium or high SCC reading compared with the low BoHV-6 cows (4/24 (16.7%) vs. 19/82 (23.2%), respectively). Metritis and endometritis are both extremely common in dairy cows, estimated to occur in around 40% and 20% of cows, respectively [34]. This was confirmed by the results of this study in which 61% of the cows were diagnosed with either metritis or endometritis, but there was no evidence supporting any association between uterine disease and the presence of either BoHV-6 or BoHV-4. On the contrary, high BoHV-6 cows had proportionally fewer cases of both uterine disease and mastitis during the first 50 days of lactation. We show here, as discussed below, that there are changes in immunity associated with BoHV-6. While this is likely to have altered the immune response of cows to other infections, an increased infection rate seems more likely, so this may have been a chance finding.

Measurements of various blood and milk metabolites did, however, show significant differences between high and low BoHV-6 score cows. Our samples were collected at around 14 DIM, when cows experience major metabolic challenges as they transition into lactation [48,49]. This commonly causes a negative energy balance (NEB) [50] that, in turn, is associated with raised circulating concentrations of NEFA and BHB and reductions in glucose and the metabolic hormone IGF-1 [49,51]. With respect to the measured metabolites, fructosamine concentrations were lower in the blood and glucose-6-phosphate was lower in milk in cows with high BoHV-6 scores. Circulating concentrations of fructosamine are thought to reflect the average plasma glucose concentration over the previous two-to-three weeks [52] and have been used to identify disturbances in glucose metabolism in a variety of species including sheep and cattle [53]. Within milk, glucose-6-phosphate reflects the nutrient availability and metabolic turnover in the mammary gland [54]. BHB was higher in the milk in our study, and elevated milk BHB concentrations can been used as an indicator of ketosis [32]. Urea concentrations were lower in both the blood and milk of high BoHV-6 cows, indicating a dietary protein deficiency. Uric acid was also lower in the milk, and this has been suggested as an indicator of microbial protein synthesis originating, in part, from ruminal digestion [29]. Circulating IGF-1 was also significantly reduced, and this is strongly associated with a poor energy balance status [55].

The metabolic profiles of the high BoHV-6 cows therefore suggested that even though they had a slightly higher BCS, they were more deficient in both energy and protein. There is mounting evidence that metabolic and disease status in early lactation are intimately linked. On the one hand, metabolic imbalance and a shortage of glucose reduce the ability of cows to mount a robust immune defense [56,57]. On the other hand, common infections such as *E. coli* mastitis can impair hepatic function and thus influence metabolic activity [58]. Herpes viruses acquired in young animals can remain latent after the initial infection and become reactivated at times of stress, mediated by increased glucocorticoid secretion [59], as occurs in cows during calving and the periparturient period [60]. In the present study, we found that the BoHV-6 scores remained stable between 14 and 35 DIM, but no pre-calving samples were taken for comparison. It is not therefore possible to determine from our data whether latent BoHV-6 infections were reactivated in cows with the worst metabolic status or whether being infected with BoHV-6 then caused the energy deficit to develop.

The leucocyte gene expression data provided clear evidence of differences in immune pathways between the high and low BoHV-6 cows. The most significant DEG was *AQP3*. This encodes aquaporin-3, primarily known for its ability to transport water and non-ionic small solutes such as urea and glycerol across cell membranes. It can also facilitate the uptake of H_2_O_2_ into mammalian cells, thus potentially playing a role in host defense mechanisms [61,62]. Three other of the most significantly up-regulated genes in the high BoHV-6 cows are involved in protein folding within the endoplasmic reticulum (ER) (*TXNDC5*, *PDIA4*, and *XBP1*). In times of high protein synthesis, the protein load may exceed the folding capacity of the ER, and misfolded proteins thus accumulate. The cellular stress causes an unfolded protein response (UPR) involving translational and transcriptional programming to help the cell return to homeostasis [63]. Herpes viruses induce a burst of synthesis of envelope glycoproteins during lytic replication and they have evolved mechanisms to use the UPR to promote their own replication and to avoid immune surveillance [64]. *XBP1* encodes a transcription factor that regulates MHC class II genes. The IRE1-XBP1 pathway is central to the UPR and is also involved in inflammatory responses to infection. XBP1 may increase the expression of viral proteins by acting as the DNA binding partner of a viral transactivator.

Fibrinogen is an important acute phase protein that plays an essential role in coagulation, and its circulating concentration increases in various inflammatory conditions of cattle [65]. The final secreted form is composed of two trimers, with each trimer made of polypeptide chains of fibrinogen alpha, beta, and gamma, encoded by *FGA*, *FGB*, and *FGG*, respectively [66]. All three of these genes were among the most up-regulated in leucocytes of the high BoHV-6 cows in terms of fold change increases. This was unexpected because the main source of secreted fibrinogen is liver hepatocytes, with little previous evidence supporting a leucocyte source. Genes encoding albumin, transferrin, and apolipoprotein A-II, major plasma proteins that are also primarily produced by the liver, were also up-regulated. Circulating albumin concentrations usually decline in the immediate postpartum period, particularly in cows with evidence of more severe inflammation [67]. Transferrin is the main blood transport protein for iron, and its receptor TFR1 is commonly used as a viral entry point into the cell [68]. Apolipoprotein A-II is the second most abundant protein contributing to high density lipoprotein particles, which are important for cholesterol transport. The leucocyte production of these various transport proteins is unlikely to be a major determinant of their circulating concentrations, so the relevance of these changes in gene expression to cow physiology remains to be determined.

Of particular interest were the DEGs encoding proteins known to be involved in viral detection and cellular responses to viral infection, which were mainly down-regulated in the high BoHV-6 cows. These included *DDX58*, which encodes a key cytoplasmic receptor that detects both positive and negative strand RNA viruses and activates a downstream signaling cascade that leads to the production of type I interferons and pro-inflammatory cytokines. This ties in with the lower expression of genes encoding both interferon response genes (*IFIT2*, *IFIT3, IFITM3, IFITM5, LOC112441484, LOC618409*, and *OAS1Z)* and cytokines (*IL1A, IL1B, IL15*, and *IL17D*). *IFIT3* (also called retinoic acid-induced gene G protein—RIGG) encodes an IFN-inducible protein that can form a cytoplasmic complex to recognize and destroy viral RNA, acting as an inhibitor of viral replication [69]. Some viruses, e.g., hepatitis C virus, can escape the antiviral functions of the interferon-induced protein with tetratricopeptide repeats (IFIT) family by suppressing the expression of the IFIT family of genes [70].

Interferons mediate a number of antiviral responses that include an ISG15 ubiquitin-like pathway and an OAS1-RNaseL pathway [70,71]. Within the present study, a number of genes encoding proteins with a potential involvement on the ubiquitin-like pathway were down-regulated in the high BoHV-6 cows. Of these, *HECW2, HERC1, HERC5, LNX1, LOC529930, MKRN3, RNF149*, and *TRIM17* all encode E3 ubiquitin ligases, key components of ISGylation. This involves the ubiquitin-like modification of proteins that may then be targeted to lysosomes for destruction or used elsewhere. During this process, the C-terminus of ISG15 is conjugated to lysine residues in the target protein following consecutive catalysis with three enzymes E1, E2, and E3 [72]. HERC5 is recognized as a major E3 ligase that is relatively non-specific, so it is able to block the IFN-mediated rise in the total level of ISGylated cellular proteins. *SKP1* encodes S-phase kinase-associated protein 1. This forms an essential component of the SCF (SKP1-CUL1-F-box protein) ubiquitin ligase complex. *UBA7* encodes ubiquitin-like modifier-activating enzyme 7. Both HERC5 and UBA7 have recognized roles in catalyzing the ISGylation of influenza A virus NS1 protein, which attenuates its virulence [73]. The OAS1 gene family (including *OAS1Z*) senses exogenous nucleic acid. *OAS1Z* encodes a protein that synthesizes 2′,5′-oligoadenylates, which regulate the early stage of viral infection by activating latent RNase L, an endoribonuclease, resulting in viral RNA degradation and the inhibition of viral replication. Polymorphisms in this gene have been associated with susceptibility to viral infections [74]. NLRP12 acts as an anti-inflammatory agent by inhibiting both canonical and non-canonical NF-kappa-B and ERK activation pathways [75]. It also functions as a checkpoint for anti-viral DDX58 activation [76].

Other immune pathways were mainly down-regulated in the high BoHV-6 cows and included antigen processing, presentation (MHC molecules), and the activation of immune response and leukocyte activation. The latter involved genes encoding proteins involved in the detection of pathogen-associated molecular patterns (PAMPs) and TLR signaling (*ACOD1, ALPK1, LYST, TARM1*, and *TIRAP*), as well as several chemokines and chemokine receptors *(CCRL2, CXCL5, CXCR1, CXCR2*, and *LOC504773). SEMA7A*, another gene of interest, encodes semaphorin7A, a secreted glycoprotein that acts as an important immune regulator. Pox viruses and Gammaherpesviruses of the *Macavirus* genus including AlHV-1 encode semaphorin 7A mimics. Viral semaphorins have been suggested to mediate immune evasion mechanisms and alter host T-cell and dendritic cell function [77].

Viral response genes that were up-regulated in high BoHV-6 cows included *XBP1* (described above), *ADA*, and *IRF4*. *ADA* encodes a DNA exonuclease. This gene is better characterized in humans, which have three adenosine deaminases, termed ADAR1-3, although ADAR3 lacks enzymatic activity [78]. ADAR1 appears to mainly function as a pro-viral factor by suppressing dsRNA-dependent antiviral molecules including MDA5, PKR, and OASes/RNase L [78]. It can, however, also display antiviral activities against a subset of viruses including paramyxoviruses and orthomyxoviruses by acting as a restriction factor to prevent their replication [79]. *IRF4* encodes interferon regulatory factor 4. This is a lymphocyte-specific transcription factor that is implicated in a number of hematological malignancies including B-cell and Hodgkin lymphoma and is highly expressed in transformed cells infected with Epstein–Barr virus, in which it promotes their proliferation [80].

## 5. Conclusions

Using analysis of leukocyte RNA-Seq data, BoHV-6 was detected in 83.5% of cows in the study. Of these, 15% were classified as carrying a high load, with a PathSeq score >200. Herpes viruses have evolved multiple strategies to evade host innate responses and facilitate host infection. The pattern of leucocyte gene expression data provides strong evidence that the presence of BoHV-6 was responsible for modifying cellular antiviral responses to promote its own survival. Dairy cows infected with BoHV-6 also had metabolic disturbances in the early postpartum period, although our data could not distinguish between cause and effect. On the one hand, the virus may cause metabolic and inflammatory changes, possibly by causing hepatic dysregulation. Alternatively, cows that are experiencing glucose and/or protein deficiency in early lactation will be under greater metabolic stress, and this may increase the likelihood that a latent BoHV-6 infection will reactivate. We found no significant increase in clinical disease in the high BoHV-6 cows: indeed, they had proportionally fewer cases of both uterine disease and mastitis during the first 50 days of lactation. At present, very little is known about the transmission of BoHV-6, either between individuals or between different ruminant populations. In light of its worldwide distribution and prevalence, more work is warranted to determine the significance of this virus to cattle production.

## Figures and Tables

**Table 1:** BoHV-6 scores (mean ± SD) for the cows in the study by country of origin.

Country	*n*	BoHV-6 Score	Min	Max	Score 1–200 (*n*)	Score > 200 (*n*)	% Cows with Score > 200
IRE	34	215 ± 269	1	1106	19	15	44%
GBR	38	48 ± 70	1	253	35	3	8%
DNK	26	84 ± 110	1	479	23	3	11%
DEU	16	155 ± 297	3	1175	13	3	19%
BEL	6	18 ± 16	1	50	6	0	0%
ITA	43	72 ± 62	3	260	42	1	2%
Total	163	104 ± 176	1	1175	138	25	15%

**Table 2:** Body weight, body condition score, dry matter intake, and milk production measurements (mean ± SD) of cows with high (>200) and low (1–200) BoHV-6 scores over the first 50 days in milk.

Parameters ^1^	*n*	High BoHV-6	*n*	Low BoHV-6	*p*-Value
Weight (Kg)	18	688.1 ± 67.6	67	664.5 ± 67.7	0.27
BCS 14 DIM	17	3.03 ± 0.23	110	2.80 ± 0.41	<0.01
BCS 35 DIM	17	2.87 ± 0.20	110	2.66 ± 0.37	0.02
BCS change 14–35 d	17	−0.16 ± 0.19	110	−0.15 ± 0.27	0.64
DMI (kg/d)	18	17.85 ± 3.95	67	19.42 ± 4.16	0.22
Milk Yield (kg/d)	18	34.13 ± 5.79	66	35.82 ± 6.26	0.35
Milk Fat (%)	18	4.68 ± 0.68	66	4.49 ± 0.72	0.26
Milk Fat Yield (kg/d)	18	1.57 ± 0.40	66	1.58 ± 0.3	0.98
Milk Protein (%)	18	3.30 ± 0.30	66	3.38 ± 0.26	0.22
Milk Protein Yield (kg/d)	18	1.11 ± 0.22	66	1.20 ± 0.24	0.28

^1^ BCS: body condition score; DIM: days in milk; DMI: dry matter intake.

**Table 3:** Metabolite data (mean ± SD) measured in blood and milk from cows with high (>200) or low (1–200) BoHV-6 scores in early lactation.

Parameters ^1^	*n*	High BoHV-6	*n*	Low BoHV-6	*p* ^2^
Blood					
BHB (mM)	24	0.79 ± 0.093	80	0.67 ± 0.064	0.126
NEFA (µekv/l)	24	856 ± 111	80	839 ± 57	0.994
Glucose (mM)	24	3.54 ± 0.140	80	3.52 ± 0.045	0.804
IGF-1 ng/ml	24	69.0 ± 9.59	85	97.0 ± 7.06	0.034
Urea (mM)	24	2.26 ± 0.87	80	2.92 ± 1.09	0.007
Cholesterol (mM)	24	2.98 ± 0.70	80	3.02 ± 0.60	0.896
Fructosamine (μM)	24	245.3 ± 25.05	78	256.3 ± 16.10	0.042
Milk					
BHB (μM)	21	81.2 ± 45.4	77	63.4 ± 39.6	0.011
Glucose-6-phosphate (mM)	21	0.15 ± 0.04	77	0.20 ± 0.07	0.002
Glucose (mM)	21	0.19 ± 0.06	77	0.22 ± 0.08	0.270
Isocitrate (mM)	21	0.17 ± 0.05	77	0.18 ± 0.04	0.370
Urea (mM)	20	1.83 ± 1.01	77	2.82 ± 1.20	0.001
Uric acid (μM)	21	132.1 ± 63.4	77	161.6 ± 54.5	0.043
LDH (units/L)	21	2.31 ± 0.91	77	3.93 ± 5.48	0.028
NAGase (units/L)	21	1.87 ± 0.79	77	2.41 ± 1.68	0.071

^1^ BHB: beta hydroxyl-butyrate; NEFA: non-esterified fatty acids; LDH: lactate dehydrogenase; and NAGase: *N*-acetyl-β-D-glucosaminidase. ^2^ Mann–Whitney test.

**Table 4:** Summary evidence for associations with BoHV-4 and uterine disease in early lactation in cows with high (>200) or low (1–200) BoHV-6 scores.

Parameter	*n*	High BoHV-6	*n*	Low BoHV-6
Total cows in group	23		89	
No. with BoHV-4 score ≥1	1		4	
% with BoHV-4		4.4%		4.5%
No. with no uterine disease	10		28	
No. with endometritis or metritis	12		47	
% with uterine disease		54.5%		62.7%
14 DIM				
Uterine discharge appearance score	22	1.5 ± 1.01	76	1.7 ± 1.13
Uterine discharge smell score	20	0.2 ± 0.41	65	0.2 ± 0.36
Ratio PMN:UEC ^1^	20	2.8 ± 5.51	69	2.7 ± 7.14
% PMN ^1^	20	17.9 ± 31.63	69	14.5 ± 35.14
35 DIM				
Uterine discharge appearance score	22	0.7 ± 0.65	84	0.5 ± 0.72
Uterine discharge smell score	19	0.0 ± 0.00	74	0.0 ± 0.16
Ratio PMN:UEC ^1^	15	0.1 ± 0.20	64	0.3 ± 0.69
% PMN ^1^	16	7.8 ± 12.83	64	13.6 ± 20.86

DIM: days in milk; PMN: polymorphonuclear neutrophil; and UEC: uterine epithelial cell. ^1^ Assessed from cytobrush smear sample.

**Table 5:** Gene expression levels in circulating leukocytes collected at around 14 days in milk compared between cows with High (H, >200) and Low (L, 1-200) BoHV-6 scores, listing the 20 most significant DEG (all up-regulated in High score cows).

Gene Symbol	Gene Name	BH ^1^ (*p*-Value)	Mean High BoHV-6	Mean Low BoHV-6	Fold Change (H/L)
*AQP3*	Aquaporin 3	0.0002	7.91	5.03	1.57
*CASC4*	Golgi membrane protein 2	0.0002	27.82	21.97	1.27
*LOC100848815*	SLA class II histocompatibility antigen, DQ haplotype D alpha chain-like	0.0002	759.3	401.6	1.89
*PERP*	p53 apoptosis effector related to PMP22	0.0003	0.43	0.23	1.86
*MZB1*	Marginal zone B and B1 cell specific protein	0.0007	83.46	47.46	1.76
*SEC11C*	SEC11 homolog C, signal peptidase complex subunit	0.0008	50.59	36.61	1.38
*RRBP1*	Ribosome binding protein 1	0.0008	24.19	18.24	1.33
*HSP90B1*	Heat shock protein 90 beta family member 1	0.0009	298.7	207.1	1.44
*IRF4*	Interferon regulatory factor 4	0.0009	48.29	36.79	1.31
*LOC100300806*	Putative V-set and immunoglobulin domain-containing-like protein IGHV4OR15-8	0.0010	93.34	66.11	1.41
*TXNDC5*	Thioredoxin domain containing 5	0.0013	61.22	39.86	1.54
*ATP7B*	ATPase copper transporting beta	0.0013	0.61	0.36	1.70
*LOC112441499*	Immunoglobulin lambda-1 light chain-like	0.0013	200.4	132.3	1.51
*PDIA4*	Protein disulfide isomerase family A member 4	0.0013	76.55	53.40	1.43
*XBP1*	X-box binding protein 1	0.0013	94.92	72.16	1.32
*CKAP4*	Cytoskeleton associated protein 4	0.0013	23.17	17.84	1.30
*KCNK1*	Potassium two pore domain channel subfamily K member 1	0.0016	6.14	4.76	1.29
*GNAI1*	G protein subunit alpha i1	0.0016	4.82	3.53	1.37
*TSPAN13*	Tetraspanin 13	0.0016	48.92	38.03	1.29
*BHLHA15*	Basic helix-loop-helix family member a15	0.0016	5.85	3.38	1.73

^1^ Analysis with Benjamini-Hochberg adjustment.

**Table 6:** Gene expression levels in circulating leukocytes collected at around 14 days in milk compared between cows with High (H, >200) and Low (L, 1-200) BoHV-6 scores, listing the Top 20 most up-regulated and down-regulated genes in High BoHV-6 cows.

Gene Symbol	Gene Name	BH ^1^ (*p*-Value)	Mean High BoHV-6	Mean Low BoHV-6	Fold Change (H/L)
Most up-regulated DEG in High BoHV-6 (based on fold changes)					
*APOA2*	Apolipoprotein A2	0.0301	0.44	0.01	31.48
*FGG*	Fibrinogen gamma chain	0.0314	0.59	0.02	24.68
*FGA*	Fibrinogen alpha chain	0.0361	0.49	0.02	24.23
*ALB*	Albumin	0.0282	3.36	0.17	20.33
*FGB*	Fibrinogen beta chain	0.0346	0.43	0.02	18.43
*TF*	Transferrin	0.0291	0.44	0.02	17.95
*FCRL6*	Fc receptor like 6	0.0232	0.45	0.12	3.66
*M1AP*	Meiosis 1 associated protein	0.0143	0.24	0.10	2.48
*PYY*	Peptide YY	0.0074	0.34	0.14	2.39
*LOC100295719*	T cell receptor gamma variable 3	0.0421	0.68	0.30	2.28
*VCAM1*	Vascular cell adhesion molecule 1	0.0291	0.26	0.12	2.13
*TNFRSF17*	TNF receptor superfamily member 17	0.0072	2.17	1.03	2.10
*SLC7A10*	Solute carrier family 7 member 10	0.0143	0.67	0.33	2.04
*LOC618565*	Natural killer cells antigen CD94	0.0267	1.34	0.66	2.03
*LOC781584*	Uncharacterised	0.0437	0.45	0.22	2.02
*MGC127055*	Uncharacterised	0.0421	0.77	0.38	2.02
*IGFBP7*	Insulin like growth factor binding protein 7	0.0125	0.60	0.30	2.01
*ALDH1A1*	Aldehyde dehydrogenase 1 family member A1	0.0137	0.79	0.41	1.93
*LOC614522*	Transmembrane protein 56	0.0024	0.31	0.16	1.92
*LOC100848815*	BOLA-DQA1	0.0002	759.3	401.6	1.89
*CD109*	CD109 molecule	0.0023	0.201	0.11	1.86
Most down-regulated DEG in High BoHV-6 (based on fold changes)					
*OR12D2*	Olfactory receptor family 12 subfamily D member 2	0.0399	0.04	0.32	−9.02
*RNF212B*	Ring finger protein 212B	0.0322	0.06	0.26	−4.48
*KCNK7*	Potassium two pore domain channel subfamily K member 7	0.0449	0.04	0.12	−2.89
*C13H20orf204*	Chromosome 13 C20orf204 homolog	0.0143	0.10	0.28	−2.64
*BOLA-DQA2*	Major histocompatibility complex, class II, DQ alpha 2	0.0072	50.82	125.7	−2.47
*VNN3*	Vanin 3	0.0217	0.55	1.33	−2.39
*ATF7IP2*	Activating transcription factor 7 interacting protein 2	0.0457	0.07	0.17	−2.36
*LOC513941*	Cationic amino acid transporter 3-like	0.0152	0.51	1.17	−2.30
*LOC112446404*	Uncharacterised	0.0118	0.22	0.51	−2.30
*LOC527385*	Cationic amino acid transporter 3-like	0.0391	0.85	1.95	−2.29
*NMUR2*	Neuromedin U receptor 2	0.0390	0.06	0.14	−2.24
*ZSWIM5*	Zinc finger SWIM-type containing 5	0.0249	0.118	0.26	−2.23
*SAMD5*	Sterile alpha motif domain containing 5	0.0438	0.089	0.19	−2.12
*ANKS1B*	Ankyrin repeat and sterile alpha motif domain containing 1B	0.0370	4.395	9.20	−2.09
*SLC22A13*	Solute carrier family 22 member 13	0.0321	0.295	0.59	−2.00
*APOBEC3Z1*	Apolipoprotein B mRNA editing enzyme, catalytic polypeptide-like 3A	0.0282	8.565	17.09	−2.00
*IGFBP1*	Insulin like growth factor binding protein 1	0.0322	0.270	0.53	−1.97
*LOC617079*	ATP-binding cassette transporter C4-like	0.0291	10.638	20.49	−1.93
*PLB1*	Phospholipase B1	0.0315	0.388	0.74	−1.92
*KEL*	Kell metallo-endopeptidase (Kell blood group)	0.0374	0.971	1.85	−1.90

^1^ Analysis with Benjamini-Hochberg adjustment.

**Table 7:** Summary of main functions of DEGs identified as having a role in immune responses between cows with high (>200) and low (1–200) BoHV-6 scores.

Function	Down-Regulated in High BoHV-6 Cows	Up-Regulated in High BoHV-6 Cows
Chemokines and their receptors	*CCRL2, CXCL5, CXCR1, CXCR2*, and *LOC504773*	*CCR10*
Cytokines and their receptors	*IL1A, IL1B, IL15, IL17D*, and *IL17RD*	*IL20RB, LOC100139916, PDGFB, TNFRSF17*
Coagulation		*FGA, FGB*, and *FGG*
Complement system	*C5AR1* and *C5AR2*	*C1QA*
Detection of pathogen-associated molecular patterns (PAMPs) and TLR signaling	*ACOD1, ALPK1, LYST, TARM1*, and *TIRAP*	
E3 ubiquitin-protein ligases	*HECW2, HERC1, HERC5, LNX1, LOC529930, MKRN3, RNF149, SKP1*, and *TRIM17*	*RNF43*
Immunoglobulins, their receptors and associated molecules	*CD101, FCGR2B, IGSF6, LOC107131323, LOC112441494, LOC112447526, OSCAR*, and *PIGR*	*CD40LG, JCHAIN, MZB1*, and *VCAM1*
MHC molecules	*BOLA-DQA2, BOLA-DQA5, LOC788634*, and *LOC100848815*	*BLA-DQB*
Regulators of immune responses	*MEFV, SLAMF6*, and *ZNF683*	
Viral detection and response including interferon-stimulated genes (ISGs)	*DDX58, DX6, IFIT2, IFIT3, IFITM3, IFITM5, LOC112441484, LOC618409,NLRP12, OAS1Z, SEMA7A*, and *UBA7*	*ADA, IRF4*, and *XBP1*
Miscellaneous	*CD22, CRISPLD2, SIGLEC5, SIGLEC8*, and *VWA3B*	*KLRG1, PLD3*, and *S100A13*

## Supplementary Materials

The following are available online at https://www.mdpi.com/1999-4915/12/12/1451/s1. Figure S1. Principal component analyses before (A,C) and after (B,D) the removal of the herd effect using the data derived from six (A,B) or four (C,D) herds. The leukocyte samples of Holstein Friesian dairy cows were taken from six farms in different EU countries at about 14 days after calving. The RNA-Seq reads were normalized with reads per kilobase of transcript per million mapped reads (RPKM). Two herds (CRA—Italy and CRA-W—Belgium) were omitted from the comparison of cows with high (>200) and low (1–200) BoHV-6 scores, as only one cow in the Italian herd had a high score. Figure S2. Principal component analysis for leukocyte transcriptomic gene expression. The RNA-Seq reads were normalized with reads per kilobase of transcript per million mapped reads (RPKM). Samples were taken from four farms in different EU countries at about 14 days after calving. Cows were classified as having a high (>200 and *n* = 90) or low (1–200 and *n* = 24) BoHV-6 score. Table S1. Complete list of DEGs in leukocytes in early lactation between cows with a high (BoHV-6 > 200) or low (BoHV-6 1–200) score with BH adjustment; *p* < 0.05 and FC ≥ 1.25.
